# Motion Adaptive Vertical Handoff in Cellular/WLAN Heterogeneous Wireless Network

**DOI:** 10.1155/2014/341038

**Published:** 2014-03-11

**Authors:** Limin Li, Lin Ma, Yubin Xu, Yunhai Fu

**Affiliations:** CRC, School of Electronics and Information Engineering, Harbin Institute of Technology, Nan Gang District, Harbin 150001, China

## Abstract

In heterogeneous wireless network, vertical handoff plays an important role for guaranteeing quality of service and overall performance of network. Conventional vertical handoff trigger schemes are mostly developed from horizontal handoff in homogeneous cellular network. Basically, they can be summarized as hysteresis-based and dwelling-timer-based algorithms, which are reliable on avoiding unnecessary handoff caused by the terminals dwelling at the edge of WLAN coverage. However, the coverage of WLAN is much smaller compared with cellular network, while the motion types of terminals can be various in a typical outdoor scenario. As a result, traditional algorithms are less effective in avoiding unnecessary handoff triggered by vehicle-borne terminals with various speeds. Besides that, hysteresis and dwelling-timer thresholds usually need to be modified to satisfy different channel environments. For solving this problem, a vertical handoff algorithm based on Q-learning is proposed in this paper. Q-learning can provide the decider with self-adaptive ability for handling the terminals' handoff requests with different motion types and channel conditions. Meanwhile, Neural Fuzzy Inference System (NFIS) is embedded to retain a continuous perception of the state space. Simulation results verify that the proposed algorithm can achieve lower unnecessary handoff probability compared with the other two conventional algorithms.

## 1. Introduction

The fourth generation wireless communication system is expected to integrate several types of different wireless technologies. Future wireless network may consist of multiple layers such as cellular, WLAN, WiMAX, and satellite. It means that subscribers may always have more than one suitable network to select according to the preference and different characteristics of each type of wireless technologies. Common trends show that WLAN is an important supplement to cellular network, because the coexisting of cellular and WLAN can bring better quality of service to subscribers. In general, cellular system performs better in the services of high mobility and low latency, while it provides lower data rate than WLAN. Therefore the interworking and cooperation of these two different types of wireless network will become an important issue in the next generation cellular system.

Common trends show that vertical handoff can help the network satisfy the diverse Quality of Service (QoS) demands from different users. It is evident that the interworking of different types of wireless network is an important issue in the heterogeneous wireless network. Unnecessary handoff, which can degenerate the overall performance of network, is one of the most detrimental issues that need to be addressed with respect to handoff mechanism. It usually occurs when mobile dwells between two base stations, and then the base stations bounce the link with the mobile back and forth. In order to solve this problem, hysteresis-based [1, 2] and dwelling-timer-based [3] handoff trigger algorithms are generally used in sole cellular network.

In recent years, many kinds of vertical handoff algorithms are proposed for reducing the effect of unnecessary handoff in heterogeneous wireless network. In 2001, Ylianttila et al. analyzed the handoff delay in GPRS/WLAN network and proposed a dwell timer based handoff algorithm to optimize the handoff decisions [4]. Afterward, McNair and Zhu proposed a handoff algorithm by comprehensively considering service type, monetary cost, network conditions, et al. [5]. In 2007, W. Lee et al. presented a movement aware vertical handoff trigger scheme for WLAN/WiMAX heterogeneous network. In this paper, an adaptive dwell timer was used to allow MS a better connection [6]. In the same year, A SINR based vertical handoff algorithm is provided by K. Yang. This algorithm converts the SINR value from one network to an equivalent value for the target network and then provides the handoff decision [7]. In addition to the above achievements, Chang proposed an adaptive vertical handoff algorithm with predictive RSS in 2008. Simulation results that validated this algorithm can reduce unnecessary handoff and improve dropping rate [8]. More recent studies were given by Haider et al. in 2011. They used intelligent fusion of adaptive threshold, signal trend, and dwell timer as the inputs of vertical handoff trigger algorithm and obtained a better result than many of traditional algorithms [9]. In [10], the authors proposed a distributed vertical handoff strategy for vehicle to vehicle and vehicle to infrastructure communication. The communication cost and transmitting time is discussed.

However these previous researches are mainly developed from the hysteresis-based and dwelling-timer-based algorithms, which are widely used in horizontal handoff of cellular network. In vertical handoff, the velocity factor has more imperative effect in handoff decision than in horizontal handoffs. The coverage of WLAN is much smaller than cellular network. Therefore, switching to WLAN when traveling at high speeds is likely to face the problem that a handoff back to the original network would occur very shortly afterwards. Since the procedure of a handoff behavior involves a set of signaling functions and consequently imposes both processing loads and signaling overhead to the network, unnecessary handoffs should be discouraged [10, 11]. Definitely, traditional algorithms are reliable on avoiding ping-pong handoff caused by the terminals dwelling at the edge of WLAN coverage, but they cannot essentially solve the unnecessary handoff triggered by vehicle-borne terminals. Moreover, hysteresis and dwelling-timer thresholds usually need to be modified to satisfy different channel environments.

Q-learning [12] is an online learning algorithm with outstanding adaptive ability. It has been widely explored in the field of automatic control. To overcome the limitations of classical Q-learning algorithm, for example, discrete state perception and discrete actions, Jouffe proposed the method that using NFIS to retain continuous perception of the state space [13]. It infers the global policy, which is relative to state, from local policies associated with each rule of the learner. NFIS is embedded to introduce generalization in the state space and generate continuous actions. On the other hand, Q-learning is used to tune a fuzzy controller. Note that, there were also some other approaches of extending fuzzy logic into Q-learning before [13] was proposed, such as those shown in [14]. In order to avoid confusion, we term the algorithm Q-learning Neural Fuzzy Inference System (Q-NFIS) in this paper instead of Fuzzy Q-learning (FQL) in [13], although it is developed from that.

The key contribution of this paper is stated as follows. Firstly, we present a Q-NFIS based vertical handoff trigger algorithm. Multiple RSS values from different AP and their rates of change were used as the input of handoff decider. It is certain that RSS values are related to the position of terminals, whereas their rates of change reflect the motion states. Consequently, the position and motion information are used as hidden function to trigger handoff decision. Then we propose an outdoor AP deployment scheme. Afterwards we analyze the unnecessary handoff triggered by vehicle-borne terminals with various speeds. Aiming at this issue, we provide the mathematical analysis for our results. Simulation results verify that the proposed algorithm can provide the decider with self-adaptive ability for handling the terminals' handoff requests with different motion types and channel conditions and achieve lower unnecessary handoff probability compared with the other two conventional algorithms. Note that many researches have proved that unnecessary handoff results in degradation of network performance [4–6, 8] with respect to throughput, delay et al. Therefore we mainly focus on how to reduce unnecessary handoff in this paper.

The scheme of this paper is organized as follows: we begin in Section 2 by discussing the mathematical model of Q-NFIS. Then we propose an AP deployment scheme for outdoor scenario in Section 3. Additionally, we derive the mathematical probability of unnecessary handoff triggered by vehicle-borne terminals with low dwelling time. In Section 4, we present the related numerical results. Meanwhile, two basic types of hysteresis-based and dwelling-timer-based handoff trigger methods are provided for comparison. Finally, Section 5 provides the conclusions of this paper.

## 2. Mathematical Model of Q-Learning Neural Fuzzy Inference System

**Figure 1:**
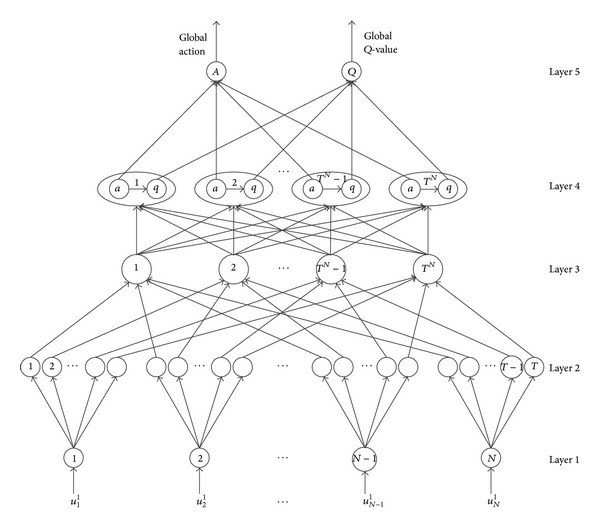
Topology of Q-learning neural fuzzy inference system.

The topology of Q-NFIS is shown in Figure 1. Essentially, Q-NFIS is a feed forward network with multiple layers. The neurons in different layers achieve different functions and are shown as follows in detail. The only information available for learning is the system feedback, which is the reinforcement signal according to the last action it has performed in the previous state. We use *u*_*i*_^*k*^ and *O*_*i*_^*k*^ to represent the input and output of the *i*th node in *k*th layer, respectively.

*Layer 1*. This layer consists of *N* neurons, which transmit the input value directly by

(1) $$\begin{array}{c} O_{i}^{1}=u_{i}^{1},\;\forall i\in \left\{1,2,\ldots ,N\right\}. \end{array}$$

In this work, we take a 4-dimensional vector as input variables, including two RSS values from different AP and their rates of change (RoC). Here we use the mean value of the RSS between the interval of current handoff request and the last one. This can reduce the deviation caused by shadow fading according to maximum likelihood estimation principle. Therefore the input can be represented by

(2) U1(t)=[u11(t),u21(t),u31(t),u41(t)]=[RSS1−(t),RSS2−(t),RoC1(t),RoC2(t)].

*Layer 2*. The function of the *T* neurons in this layer is fuzzification of input value. As shown in (3) and (4), *ℳ*(·) is the linguistic variable related to input value; namely, *O*_*i*_^2^ is the fuzzy membership value with respect to input. It reflects the degree that input value corresponds with *ℳ*(·). Gaussian Function is used as the parameterized membership function and the relationship between input and output is shown in (4). Each row in matrix **M** ^*N*×*T*^ is a linguistic variable set related to one dimension of the input state vector. The linguistic variable set should cover the entire area with respect to the possible distribution of the input and then each possible input case can be described precisely.

Consider the following:

(3) Oi2=MN×T=[ℳ1,1(u12)⋯ℳ1,T(u12)⋮⋱⋮ℳN,1(uN2)⋯ℳN,T(uN2)],

(4) ℳi,j(ui2)=exp⁡⁡(−12(ui2−mi,jσi,j)2),∀i∈{1,2,…,N};  ∀j∈{1,2…,T}.

*Layer 3*. Achieve the fusion of fuzzy rules, which equal to fuzzy multiplication operation by each input value. Define *u*_*i*,*j*_^3^ as the input from *j*th membership of *O*_*i*_^1^, and then we can obtain the strength of each rule by

(5) Ok3=∏iui,j3=∏i({ℳi,ji(ui2) ∣ ∀ℳi,ji(ui2)∈Mi})∀i∈{1,2,…,N},  ∀j∈{1,2,…,T},  ∀k∈{1,2,…,TN}.

*Layer 4*. Every neuron in this layer includes a local action-reward pair which is represented as (*a*_*i*_, *q*_*i*_). Global action and global 𝒬 value are obtained by the fusion of local action and local *q* value, respectively. Local action is a finite set of output space predefined by system. With regard to a vertical handoff decider, the local action set can be defined as *𝔸* = {Reject(*R*), Access(*A*)}.

The local action *a*_*i*_ ∈ *𝔸* is guided by the related local reward *q*(*φ*_*i*_, *a*_*i*_). Assume that the optimal local action is *a*_i_*, which satisfies

(6) $$\begin{array}{c} a_{i}^{*}=\underset{a_{i}}{\text{argmax}}\left\{q\left(\varphi_{i},a_{i}\right)\right\},\;{\forall a}_{i}\in 𝔸,\;\;\forall i\in \left\{1,2,\ldots ,T^{N}\right\}. \end{array}$$

As shown in (7), the output of Layer 4 is the normalized local action and *q* value, respectively. *α*_*i*_(*x*) is the rule set and *q*(*φ*_*i*_, *a*_*i*_) is the *q* value of state-action pair (*φ*_*i*_, *a*_*i*_).

Consider the following:

(7) $$\begin{array}{c} O_{i}^{4}=\frac{\alpha_{i}\left(x\right)\cdot a_{i}^{*}}{\sum_{i=1}^{T^{N}}\alpha_{i}\left(x\right)},\\ {\hat{O}}_{i}^{4}=\frac{\alpha_{i}\left(x\right)\cdot q\left(\varphi_{i},a_{i}^{*}\right)}{\sum_{i=1}^{T^{N}}\alpha_{i}\left(x\right)}. \end{array}$$

*Layer 5*. Achieve the function of defuzzy by linear summation of local action and local *q* value, respectively.

Consider the following:

(8) $$\begin{array}{c} O_{i}^{5}={𝒜}^{*}\left(U^{1}\left(t\right)\right)=\overset{T^{N}}{\underset{i=1}{\sum }}u_{i}^{5},\\ {\hat{O}}_{i}^{5}=𝒬\left(U^{1}\left(t\right),{𝒜}^{*}\left(U^{1}\left(t\right)\right)\right)=\overset{T^{N}}{\underset{i=1}{\sum }}{\hat{u}}_{i}^{5}. \end{array}$$

Consider that the state transition is $\left(U^{1}\left(t\right),{𝒜}^{*}\left(U^{1}\left(t\right)\right)\right)\overset{\text{handoff}}{\to }U^{1}\left(t+\tau_{l}\right)$. According to the update principle from classical Q-learning [12], the global 𝒬 value is updated by (9), where *α* is learning rate, *β* is discount factor, *𝓇* is reward value, and *τ*_*l*_ is the handoff latency.

Consider the following:

(9) 𝒬′(U1(t),𝒜∗(U1(t))) =(1−α)𝒬(U1(t),𝒜∗(U1(t)))  +α(𝓇+β𝒬(U1(t+τl),𝒜∗(U1(t+τl)))).

Then we can get the difference of 𝒬 value by

(10) Δ𝒬=𝒬′(U1(t),𝒜∗(U1(t)))−𝒬(U1(t),𝒜∗(U1(t)))=α(r−𝒬(U1(t),𝒜∗(U1(t)))+β𝒬(U1(t+τl),𝒜∗(U1(t+τl)))).

Afterward, local *q* value can be updated according to the global 𝒬 value by

(11) $$\begin{array}{c} q^{\prime }\left(\varphi_{i},a_{i}^{*}\right)=q\left(\varphi_{i},a_{i}^{*}\right)+\Delta q=q\left(\varphi_{i},a_{i}^{*}\right)+\frac{\Delta 𝒬\cdot \alpha_{i}\left(x\right)}{\sum_{i=1}^{T^{N}}\alpha_{i}\left(x\right)}. \end{array}$$

After terminal logging out, reward value in the form of positive or negative is evaluated according to the validity of the handoff decision and applied to tune local *q* value. Considering reward value reflects the evaluation to the handoff decision, it should be set as the normalized values which reflect the system performance as expected. For example, if low unnecessary handoff is required, the absolute value of reward in case 1 should be set bigger than that of the other three cases referring to Table 1. As a result, access policy becomes stricter and wrong reject rate in case 3 will grow as tradeoff.

## 3. Mathematical Analysis of Motion Model and Simulation Scenarion

### 3.1. Simulation Scenario

**Figure 2:**
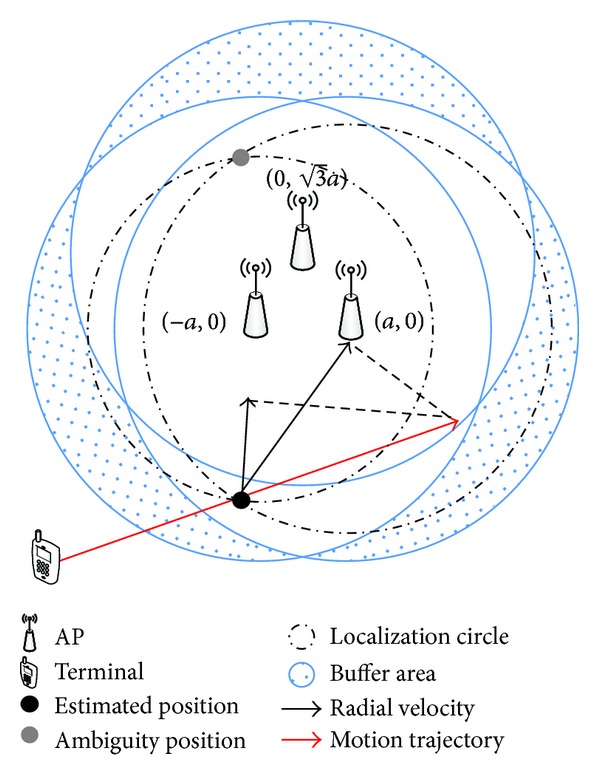
An outdoor AP deployment scheme.

Inspired by the structure of cellular network, we propose an outdoor AP deployment scheme in this work. As shown in Figure 2, each three APs constitute a cluster. The advantage is that terminals are covered by more than one AP at most of the area. Therefore, they can receive more than one dimension of beacon signal, which can be used as the reference of handoff decision. Moreover, this structure can enhance the communication capacity for hotspot area. Referring to Figure 2, shadow region covered by single AP is regarded as buffer area, which is similar to hysteresis-based methods. It can reduce the unnecessary handoff triggered by terminals dwelling at the edge of WLAN coverage. We take a 4-dimensional vector as input variables, including two RSS values from different AP and their rates of change. For getting the changing rate of RSS, the handoff controller starts to work after collecting at least 2 sets of RSS at different time points. A typical log-normal propagation model of WLAN signal as (12) is adopted in this simulation.

Consider the following:

(12) $$\begin{array}{c} P\left(d\right)=P_{t}-P_{0}-10\gamma \log_{10}\left(d\right)+\varepsilon \left(\mu ,\sigma \right). \end{array}$$

### 3.2. Motion Model

**Figure 3:**
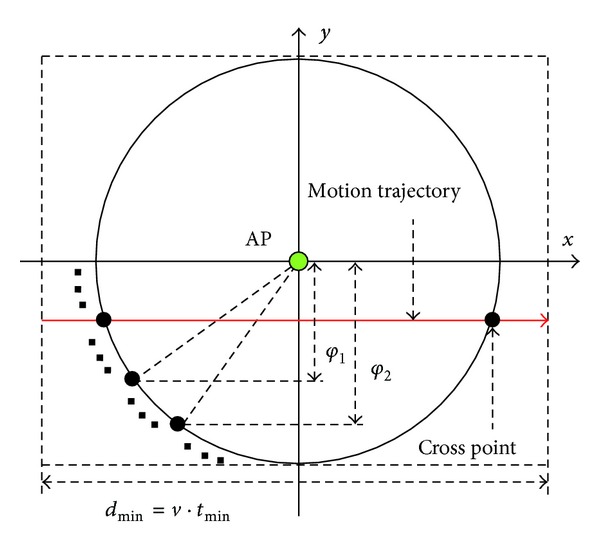
Schematic diagram of terminal moving through WLAN coverage.

The substantial idea of this paper is using Q-NFIS to estimate the terminal's motion information and then giving the handoff decision by predicting whether unnecessary handoff will be triggered. However, prediction is not always reasonable in real condition because terminal will not keep a same motion state all the time. The confidence of the handoff decision given by Q-NFIS relates to not only the performance of the algorithm itself but also the probability that terminals keep the stable motion state during the duration dwelling in WLAN coverage. Therefore this issue is analyzed based on a conditional random walk model for supporting the reasonability of our algorithm in this section. In addition, we derive an approximate estimation about the proportion of unnecessary handoff that can be predicted theoretically. Assuming the terminal's motion in accordance with random walk model, the probability of different angles that terminal moves into the coverage is the same. Therefore we only need to consider the case that terminal moves into the coverage of WLAN as horizontal direction, as shown in Figure 3.

Since the terminal cannot change velocity frequently in a real case, we may as well assume that terminals will keep a fixed velocity for *t*_min⁡_ seconds at least. Based on the prerequisite, the trajectory will be *d*_min⁡_ = *vt*_min⁡_ with fixed velocity. Assuming that *r* is the radius of WLAN coverage, *p*_*fx*_ and *p*_*ufx*_ are the probability that terminals pass through the coverage of WLAN with fixed and unfixed velocity, respectively. According to the different conditions of *d*_min⁡_ ≥ 2*r* and *d*_min⁡_ ≤ 2*r*, *p*_*fx*_ and *p*_*ufx*_ can be obtained as follows.

(i) Case 1, *d*_min⁡_ ≥ 2*r*:

(13) pufxpfx=∫0r2(r2−φ2)1/2dφ∫0r(dmin⁡/2−(r2−φ2)1/2)dφ=2∫0r(r2−φ2)1/2dφrd/2−∫0r(r2−φ2)1/2dφ.

The integration term ∫_0_^*r*^(*r* ^2^−*φ* ^2^)^1/2^ *dφ* is given by

(14) ∫0r(r2−x2)1/2dx=(x2(r2−x2)1/2+r22sin−1⁡xr)|0r=14πr2.

Substitution of (14) into (13) leads to

(15) $$\begin{array}{c} \frac{p_{ufx}}{p_{fx}}=\frac{2\pi r}{2d_{\min}-\pi r}. \end{array}$$

Considering that *p*_*fx*_ + *p*_*ufx*_ = 1, we can obtain

(16) $$\begin{array}{c} p_{fx}=\frac{{2d}_{\min}-\pi r}{2d_{\min}+\pi r}. \end{array}$$

(ii) Case 2,  *d*_min⁡_ < 2*r*.

Similar to Case 1, we can derive the result in (17), where $\Delta =\sqrt{r^{2}-{\left(d_{\min}/2\right)}^{2}}$.

Consider the following:

(17) $$\begin{array}{c} \frac{p_{ufx}}{p_{fx}}=\frac{3\Delta d_{\min}+2r^{2}\tan^{-1}\left(d_{\min}/2\Delta \right)}{2rd_{\min}-\Delta d_{\min}-2r^{2}\tan^{-1}\left(d_{\min}/2\Delta \right)},\\ p_{fx}=\frac{2rd_{\min}-\Delta d_{\min}-2r^{2}\tan^{-1}\left(d_{\min}/2\Delta \right)}{2d_{\min}\left(r+\Delta \right)}. \end{array}$$

According to the equations above, we can obtain *p*_*fx*_, which is the probability of that terminals passing through the coverage of WLAN with fixed velocity. In other words, the motion information is reliable for handoff decision by probability of *p*_*fx*_. Monte Carlo simulations were done to confirm the validity of the results.

## 4. Simulation Results and Analysis

Simulation scenario is shown in Figure 2. Relative parameters can be found in Table 2. As shown in Figure 2, two localization solutions will be obtained according to the propagation model when terminal is covered by two APs. Note that the ambiguity solution is always covered by three APs from the structure of WLAN coverage. Therefore, from the theoretical point of view, 2-dimensional RSS can reflect the location information to a certain degree when terminal implements a continuous motion state.

The motion of terminals is defined in accordance with conditional random walk model which is analyzed in Section 2, and *t*_min⁡_ is set for 1 minute. In addition, we consider three typical motion types, which are pedestrian-borne terminal, bicycle-borne terminal, and vehicle-borne terminal with the velocity of 1 m/s, 3 m/s, and 15 m/s, respectively. They are all generated outside the coverage of WLAN at a random initial position. The trigger threshold of unnecessary handoff is set for 10 seconds. For referring to the performance of Q-NFIS based handoff trigger algorithm, two basic types of hysteresis-based and dwelling-timer-based handoff trigger methods are provided for comparison in the same scenario. In the hysteresis-based handoff scheme, handoff is triggered when any dimension of RSS is greater than (*P*_*th*_ + 5) dBm. In the dwelling-timer-based handoff scheme, handoff is triggered after receiving 5 consecutive signals greater than *P*_*th*_ dBm.

**Figure 4:**
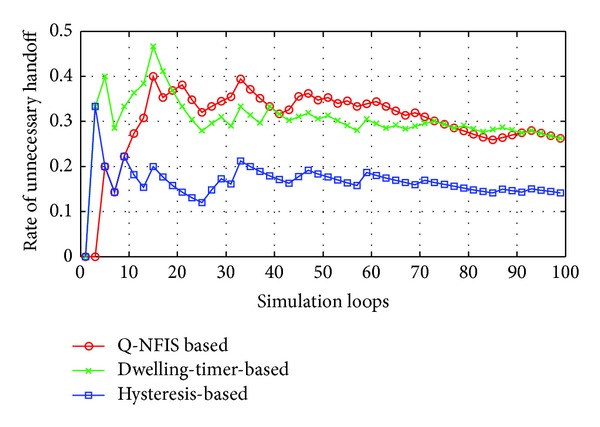
Comparison of unnecessary handoff rate for 3 algorithms in first 100 simulation loops.

**Figure 5:**
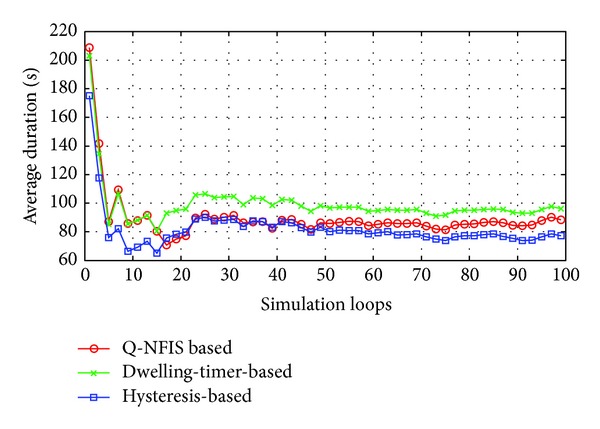
Comparison of average duration for 3 algorithms in first 100 simulation loops.

**Figure 6:**
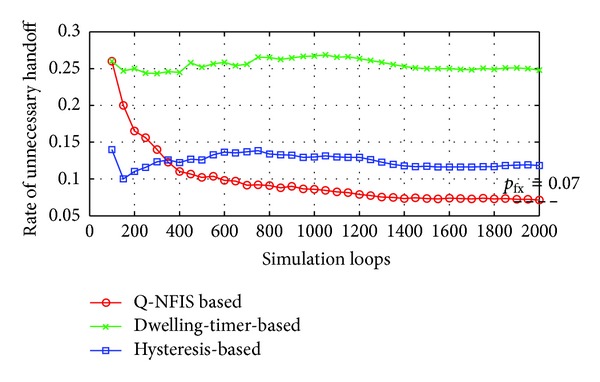
Comparison of unnecessary handoff rate for 3 algorithms in 2000 simulation loops.

**Figure 7:**
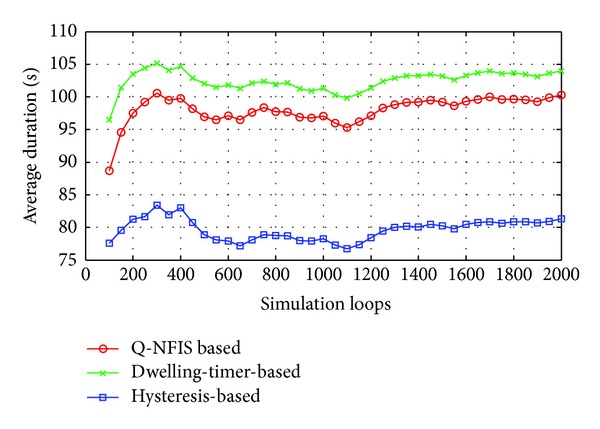
Comparison of average duration for 3 algorithms in 2000 simulation loops.

Hysteresis and dwelling-timer based algorithms are both achieved at the cost of coverage essentially, which will reduce the average service time of users acquired. Similarly, the proposed algorithm also needs a buffer period to estimate the motion state. Based on the consideration above, besides the trigger rate of unnecessary handoff, we take users' average duration of accessing WLAN into account as well. In order to present the detailed tuning trends, the simulation results of first 100 loops are shown in Figures 4 and 5, which are separated from the overall performance in Figures 6 and 7, respectively.

None priori knowledge is added into Q-NFIS; therefore the trigger rate of unnecessary handoff is high at the beginning of simulation referring to Figure 4, while terminals' average duration is low referring to Figure 5. With the growing of simulation loops, Q-learning system is tuned by collecting more and more knowledge of state space state/action pair, and the improving of the performance from Figure 7 validates the online learning ability of the proposed algorithm. As we know, unnecessary handoff is mainly triggered by vehicle-borne terminals with low dwelling time in WLAN. According to the discussion in the previous section, we can obtain that the trigger rate of unnecessary handoff by optimal handoff control is *p*_*ufx*_ approximately. From Figure 7, we can find that the simulation result by Q-NFIS is close to the optimal solution theoretically. From the simulation results shown in Figures 6 and 7, an acceptable rate of unnecessary handoff rate can be achieved by hysteresis based algorithm; however the average duration is much lower compared with the other two algorithms. It is because hysteresis costs much WLAN coverage for the characteristic of its transmission model. By dwelling-timer based algorithms we can achieve a high average duration, because it will permit handoff requests as soon as the dwelling-timer is reached. This scheme is especially good for pedestrian type terminals; however, it will be less effective for vehicle-borne terminals. This is the reason why its rate of unnecessary handoff is much higher than the other two algorithms, and meanwhile it validates the discussion we present in Section 1.

As a result, we can find that unnecessary handoff rate of Q-NFIS reduced evidently with the growing of simulation loops and nearly converged at about 0.07 after 1500 simulation loops from Figure 6. Figure 7 shows that Q-NFIS achieves terminals' average duration higher than hysteresis based scheme while a little lower than dwelling-timer based scheme. This indicates that the proposed algorithm can provide reasonable motion predictions for handoff decision by sacrificing a little degree of duration.

## 5. Conclusions

In this paper, in order to solve the problem of unnecessary handoff caused by vehicle terminals with low dwelling time, a motion adaptive vertical handoff algorithm based on Q-NFIS is proposed. For supporting the reasonability of our algorithm, we provide the mathematical analysis about the unnecessary handoff that can be predicted theoretically. Simulation results validate the proposed that algorithm can reduce unnecessary handoff effectively by providing reasonable motion predictions for handoff decision. In addition, its performance outperforms two typical traditional handoff trigger algorithms in the same simulation scenario. Therefore we can draw the conclusion that it is a reasonable vertical handoff algorithm for cellular/WLAN heterogeneous wireless network.

## Figures and Tables

**Table 1:** Reward scheme of Q-NFIS.

Case	Whether permit handoff request	Whether trigger unnecessary handoff	Reward value: positive (P), negative (N)
1	Y	Y	N
2	Y	N	P
3	N	Y	P
4	N	N	N

**Table 2:** Simulation parameters.

Parameters	Value	Description
*P*_*t*_	20 mW	Transmit power of AP
*P*_0_	37.3 dB	Path loss in the first meter
*P*_*th*_	−85 dBm	Sensitivity of terminal
*γ*	3.3	Path loss exponent
*ε*(*μ*, *σ*)	*μ* = 0, *σ* = 5	Gaussian random noise
*a*	20 m	Parameter of AP location
